# Depression Partially Mediates the Associations of Loneliness with Memory, Executive Function and Processing Speed

**DOI:** 10.1093/geroni/igaf122.3746

**Published:** 2025-12-31

**Authors:** Carmen Jia-Wen Chek, Carlos Araujo-Menendez, Ariana Stickel, Wassim Tarraf

**Affiliations:** Wayne State University, Detroit, Michigan, United States; San Diego State University/University of California, San Diego, San Diego, California, United States; San Diego State University, San Diego, California, United States; Wayne State University, Detroit, Michigan, United States

## Abstract

Loneliness and depression are linked to poorer cognition in older adults. While prior research has often examined loneliness as a mediator between depression and cognition, few studies have investigated depression as a mediator between loneliness and specific domains of cognition (e.g., memory vs. executive). This study addresses that gap by examining whether depression mediates the relationship between loneliness and memory, executive functioning, and attention/processing speed using structural equation modeling (SEM). Cognitive domains were derived using confirmatory factor analysis from the 2016 Harmonized Cognitive Assessment Protocol dataset of the Health and Retirement Study (n = 3,293; Mage = 72.71, SDage = 7.47), with loneliness (UCLA 11-item) and depression (CESD-8) from Waves 2012/2014 were tested in an SEM mediation model. Sociodemographic and physical health covariates were added stepwise. Loneliness significantly predicted increased depressive symptoms (β = 0.39), and had significant direct effects on all cognitive domains, except for memory in a fully adjusted model (β = -0.04, p = .06). Depression significantly predicted worse cognitive outcomes across all domains. Significant indirect effects on memory (β = -0.05), executive functioning (β = -0.09), and attention/ processing speed (β = -0.08) indicated partial mediation by depression. These indirect effects remained significant after covariate adjustment, with direct effects being slightly attenuated (p < .01). Findings highlight that depression only partially accounts for the cognitive risks introduced by loneliness. Loneliness presents a multi-pathway risk for cognitive decline, warranting direct intervention beyond its socioemotional effects.

